# SARS-CoV-2 Causes a Different Cytokine Response Compared to Other Cytokine Storm-Causing Respiratory Viruses in Severely Ill Patients

**DOI:** 10.3389/fimmu.2021.629193

**Published:** 2021-03-01

**Authors:** Marton Olbei, Isabelle Hautefort, Dezso Modos, Agatha Treveil, Martina Poletti, Lejla Gul, Claire D. Shannon-Lowe, Tamas Korcsmaros

**Affiliations:** ^1^Earlham Institute, Norwich, United Kingdom; ^2^Gut Microbes and Health Programme, Quadram Institute Bioscience, Norwich, United Kingdom; ^3^Institute of Immunology and Immunotherapy, The University of Birmingham, Birmingham, United Kingdom

**Keywords:** SARS-CoV-2, cytokine response, influenza A, MERS- and SARS-CoV, literature analysis, systematic review

## Abstract

Hyper-induction of pro-inflammatory cytokines, also known as a cytokine storm or cytokine release syndrome (CRS), is one of the key aspects of the currently ongoing SARS-CoV-2 pandemic. This process occurs when a large number of innate and adaptive immune cells activate and start producing pro-inflammatory cytokines, establishing an exacerbated feedback loop of inflammation. It is one of the factors contributing to the mortality observed with coronavirus 2019 (COVID-19) for a subgroup of patients. CRS is not unique to the SARS-CoV-2 infection; it was prevalent in most of the major human coronavirus and influenza A subtype outbreaks of the past two decades (H5N1, SARS-CoV, MERS-CoV, and H7N9). With a comprehensive literature search, we collected changing the cytokine levels from patients upon infection with the viral pathogens mentioned above. We analyzed published patient data to highlight the conserved and unique cytokine responses caused by these viruses. Our curation indicates that the cytokine response induced by SARS-CoV-2 is different compared to other CRS-causing respiratory viruses, as SARS-CoV-2 does not always induce specific cytokines like other coronaviruses or influenza do, such as IL-2, IL-10, IL-4, or IL-5. Comparing the collated cytokine responses caused by the analyzed viruses highlights a SARS-CoV-2-specific dysregulation of the type-I interferon (IFN) response and its downstream cytokine signatures. The map of responses gathered in this study could help specialists identify interventions that alleviate CRS in different diseases and evaluate whether they could be used in the COVID-19 cases.

## Introduction

The current coronavirus 2019 (COVID-19) pandemic has focused its attention on viral infectious diseases that the host antiviral immune response is unable to resolve. Major efforts are now concentrating on how severe acute respiratory syndrome β-coronavirus 2 (SARS-CoV-2) alters normal antiviral immune responses (1–3). SARS-CoV-2 causes a wide range of clinical symptoms from asymptomatic, through mild fever, persistent cough, loss of taste and smell, to severe inflammation-driven pneumonia, resulting in multiple organ failure and ultimately death (4–6). SARS-CoV-2 induces an anti-inflammatory response attacking both the upper and lower respiratory tracts (7, 8). Although SARS-CoV-2 appears to modify host inflammatory defenses, similar modifications are also observed in other severe respiratory infections caused by viruses such as influenza A, β-coronaviruses SARS-CoV and MERS-CoV (9–11). These agents all constitute a global health threat with colossal economic consequences (12, 13).

Although these different viruses cause similar clinical symptoms, the pathogenesis may be driven by different triggers. Multiple studies have described an increase in the pro-inflammatory host immune response associated with severe forms of the diseases, including cytokine storms or cytokine release syndrome (CRS) (11, 14, 15). Although CRS usually resolves following completion of the antiviral response, it persists in severe cases (16). It can lead to tissue damage, multiple organ failure and death in critically-ill patients if the clinical intervention is not rapid (17, 18). In such cases, concentrations of both pro- and anti-inflammatory cytokines are significantly increased in blood and other tissues, including the type-I interferons (IFNs) (IFN-α, -β, -κ, -ε, -τ, -ω, and -ζ) (19–22). Type-I IFN signaling cascades also attenuate inflammation to avoid tissue damage during viral infection (23). The main effectors of the type-I IFN signaling are IFN-α and IFN-β, which activate other cytokines, such as IL-12 and the type-II IFN cytokine, IFN-γ (24, 25). However, cytokines such as IL-10 block the type-I IFN response. Certain pathogens, including SARS-CoV and MERS-CoV, encode proteins that can influence and delay the type-I IFN response leading to various pathologies (26–28). In the case of SARS-CoV, the build-up of activated macrophages in the lungs can cause tissue damage, while MERS-CoV can intensify engagement by neutrophils, leading to an increase in the production of pro-inflammatory cytokines (29–32). Furthermore, influenza A and coronavirus infections can trigger increased levels of type-I IFN-α and IFN-β, reflecting the normal initiation of this signaling pathway in response to viral infections (33–36). However, in severe infections with SARS-CoV-2, the type-I IFN signaling is impaired, culminating in an altered development of adaptive immunity (15, 37–39).

The similar clinical symptoms and the range of disease severity of different respiratory viral infections tend to blur the accuracy of the initial diagnosis (40, 41). Capturing a clear picture of the immune response triggered in each patient, early enough in infection remains challenging. It impairs the prevention of the severe form of the disease and, consequently, the potential onset of CRS. Defining the overlap and/or specificity in the patient immune cytokine signaling across CRS-causing viruses would help clinicians to develop a more tailored treatment strategy for future cases. Recent reviews have attempted to compare diseases caused by influenza A and β-coronaviruses (42–45). To provide mechanistic insight into the role of pro- and anti-inflammatory cytokines in the development of severe diseases caused by SARS-CoV, SARS-CoV-2, MERS-CoV, and influenza viruses, understanding the differences in cytokine responses between the different viruses is vital.

To identify the similarities and differences in the cytokine response, we collected and analyzed the patterns of cytokine changes caused by these CRS-causing respiratory viruses. By comparing available patient data from the literature, we were able to show (i) where similarities lie between the immune responses mounted against these pathogens, (ii) the differences between influenza A subtypes and coronaviruses and (iii) the unique aspects of the currently circulating SARS-CoV-2 virus.

## Methods

### Literature Search

**Figure 1:**
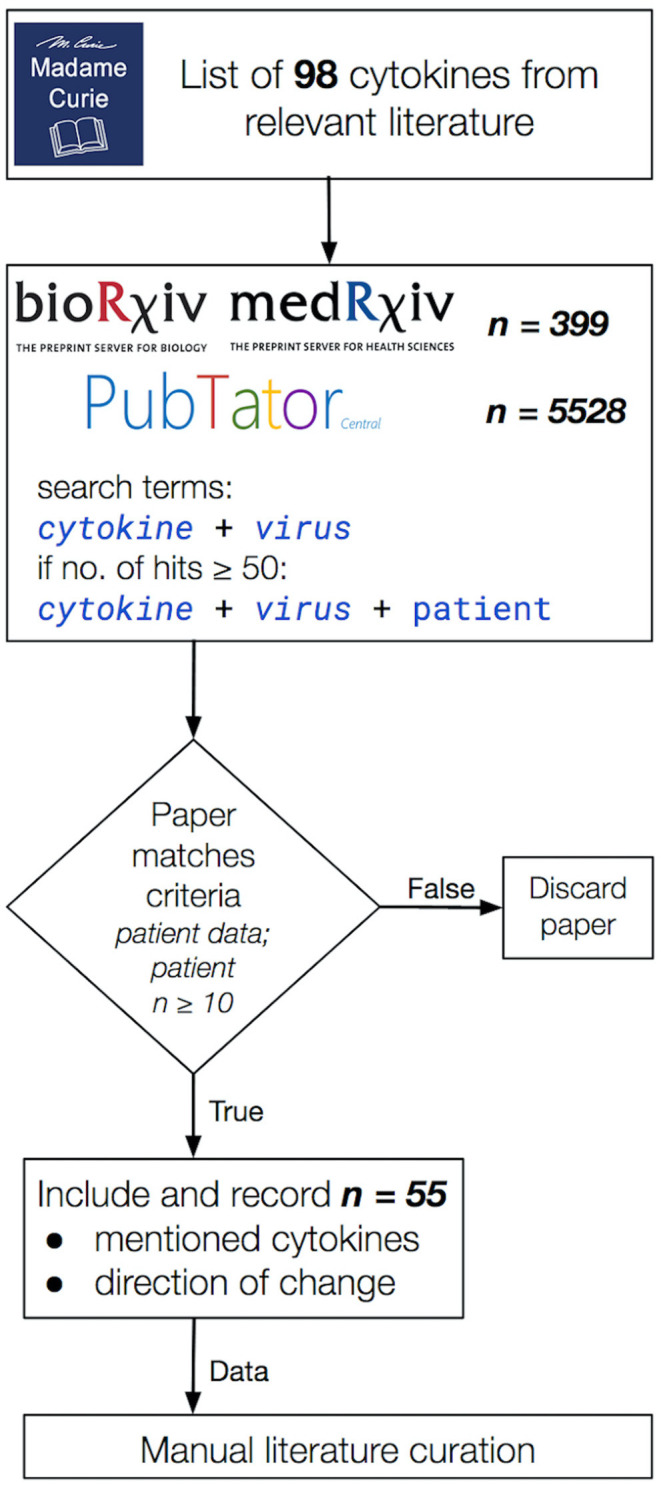
The literature curation workflow applied in this study. Publications were considered valid for the inclusion into our data collection if (i) they contained patient-derived data (model organisms and cell lines were excluded), (ii) the study data were collected from cohorts of at least 10 participants per group and (iii) it included a directional change in cytokine levels. Total hits to queries in bioRxiv, medRxiv, and PubTator are shown separately in the second box from the top. In the end, 55 publications were selected that matched our curation criteria listed above.

A mass literature search of 98 cytokines (46) was performed in PubMed using PubTator and in bioRxiv (https://www.biorxiv.org/) and medRxiv (https://www.medrxiv.org/) non-peer reviewed pre-publication repositories (47). This included the commonly studied interleukins, IFNs, tumor growth factors and chemokines involved in pro-inflammatory and anti-inflammatory responses, in particular, those associated with disease-associated CRS manifestations. Only studies indicating increase or no change in cytokine levels were included. The amplitude of change was not measured, only the presence or absence of it. We focused our study on five important CRS-causing viruses: two influenza A virus subtypes, H5N1 and H7N9, and three β-coronaviruses, SARS-CoV, MERS-CoV, and SARS-CoV-2 (Figure 1). We used the names of each virus and the cytokines and chemokines as search terms, e.g., “SARS-CoV-2 + CXCL10” (Figure 1). The collected studies were then screened to retain the studies using only patient-derived data, measured in at least 10 patients. A second pass was done adding “patient” to the search terms, e.g., “SARS-CoV-2 + CXCL10 + patient” in cases where the original search term yielded more than 50 hits. We only considered articles valid if they contained patient-derived data directly; the cell line or model organism-based results (and reviews) were excluded. From the main text of the resulting articles, we generated a table containing the presence of the queried cytokines and their direction of change in each disease. We closed the curation on March 06, 2020 (See Supplementary Table 2 for the full list of queried cytokines). A script to generate the search URLs can be found in the publication of GitHub repository (https://github.com/korcsmarosgroup/CRS). The amount of discarded articles was estimated using custom python and shell scripts, also available in the publication repository.

### Hierarchical Clustering

We clustered our data using the clustermap function from the python package seaborn with Jaccard distance and the complete linkage method (48). Jaccard distance calculates the distance between two sets of objects (49). Complete linkage clustering means that the distance from one cluster to another is calculated based on the furthest members of the cluster (50). The used clustering is sensitive for the furthest elements. Complete linkage does not join together with the furthest clusters, producing a clear picture. It performs well for finding the correct clusters in synthetic studies (51). We used all cytokine categories as input. The code is available at our GitHub repository (https://github.com/korcsmarosgroup/CRS).

## Results

In order to capture the breadth of the relevant published literature, we based our curation on a list of cytokines from the book chapter titled “Cytokines, Chemokines and Their Receptors” of the Madame Curie Bioscience Database (46) (Figure 1). We only used studies that reported the directional change of measured cytokines. Our curation approach allowed us to highlight shared and differing cytokine responses between influenza A and β-coronaviruses, contributing to further the understanding of why SARS-CoV-2 in particular differs so much not only from influenza A CRS-causing viruses but also from other β-coronaviruses, also capable of inducing a cytokine storm in severe cases.

### β-coronaviruses and Influenza A Viruses Show Marked Differences in Some Cytokine Responses

**Figure 2:**
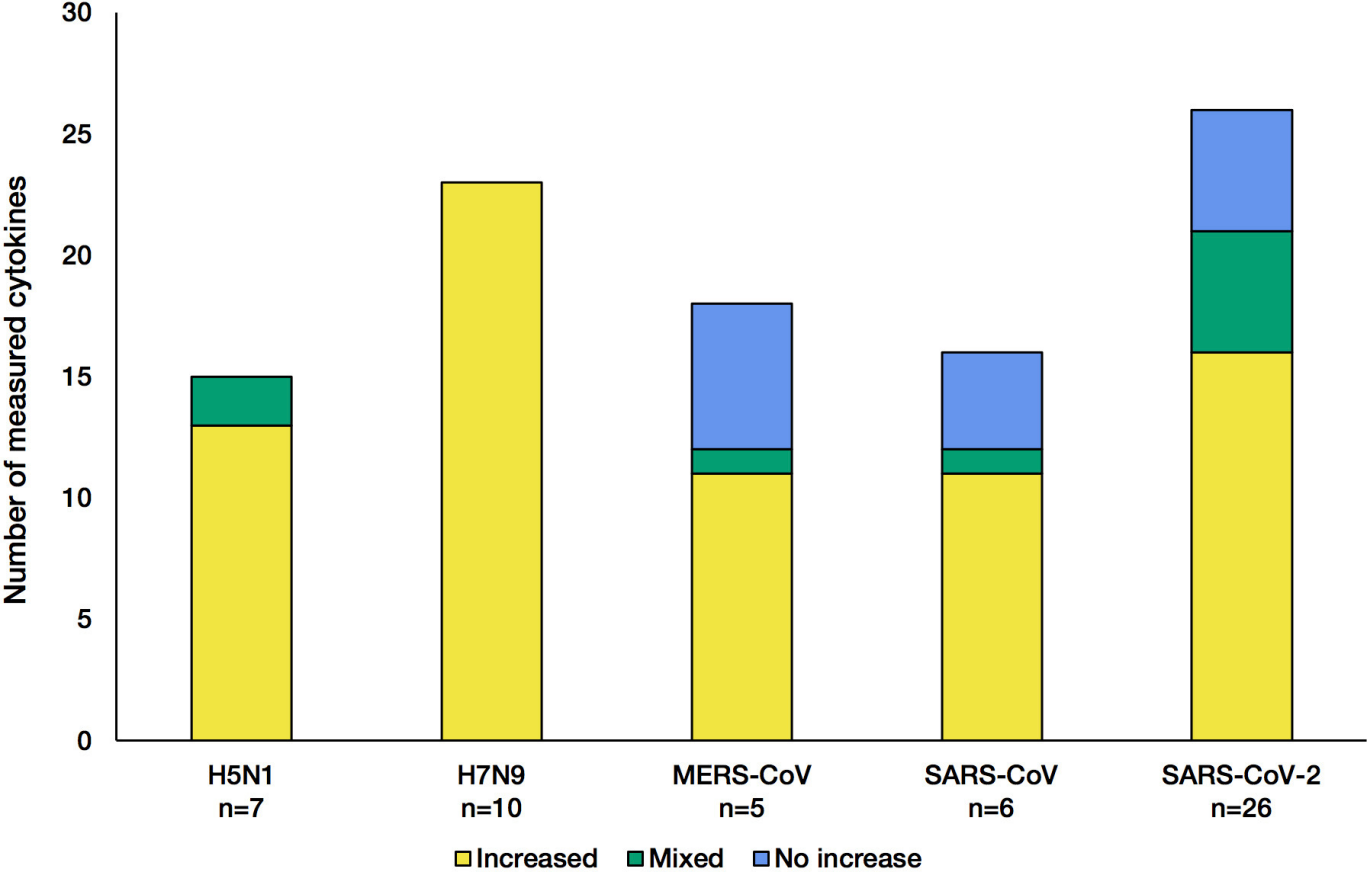
Number of cytokines measured in the studies for each of the five CRS-causing viruses. Each stacked bar indicates how many cytokines were found at increased levels (yellow) in the blood/solid tissue of the patients, not changed (blue) or both increased and not changed across different studies of the same virus (green). The *n* number shown at the bottom of the bar charts corresponds to the number of articles citing cytokine changes during infection.

Out of the nearly 100 cytokines measured across all initially-collected studies, only 38 were retained as they matched our criteria (See Methods section; Supplementary Table 1). Only a small group of cytokines was commonly measured for all viruses (CXCL8, IL-6, CXCL10, IL-2, IL-10, IFN-γ, and TNF-α). Across the 55 literature references used here (Figure 1), we first assessed how comparable the number of different cytokines measured in these studies was across the five CRS-causing viruses. Figure 2 shows how variable this number is between virus-specific studies (e.g., 15 for H5N1 and 26 for SARS-CoV-2). This variation reflects (i) the increasing interest developed for CRS-causing pathologies over recent years (26 recent studies reported cytokine measurement for SARS-CoV-2 against only 10 H5N1-related studies) and (ii) the increased availability and sensitivity of the multiplex detection method.

The influenza A viruses trigger an increase in all cytokine levels measured (Figure 2, yellow). In contrast, during infection with each of the β-coronaviruses, some cytokines were detected at levels normally found in control groups (blue). This indicates that β-coronaviruses can subvert the immune response, reflecting different kinetics and pathogenesis between the influenza- and coronavirus-associated diseases. Of note, studies of H5N1 infections showed that a few cytokines were increased compared with control groups, and no change was observed in other studies (36, 52), illustrating the greater complexity of these diseases, probably due to the multifactorial nature of the mechanisms involved.

Table 1 shows the number of cytokines whose levels are increasing in one, two, three, four or all five virus-related infections from the interrogated literature. Only five cytokines were modulated regardless of the virus-associated disease concerned, with 20 other cytokines being shared to some degree. Increased levels observed in 16 cytokines were unique to a single virus at a time. It is important to keep in mind that the amplitude of change in the cytokines is not considered, which can be different between the different diseases, adding to the heterogeneity of those severe respiratory infectious diseases. This backs up the highly complex nature of the associated diseases as well as the past and current struggles to develop efficient vaccines and treatments.

**Table 1:** Number of cytokines which were elevated in at least one study.

	**Cytokines elevated at least in one study**
	**(elevated and mixed)**
Virus-specific	16
Shared between 2 viruses	5
Shared between 3 viruses	8
Shared between 4 viruses	2
Common to all 5 viruses	5

*Cytokines measured in one or more of the virus-induced infections. Column 2 indicates the number of elevated or mixed measurements, and their overlap between viruses. Mixed observations occur when one or more studies show no change in a cytokine level upon infection, whereas others show an increase*.

To examine the presence of the measured cytokines and directionality of their change, we constructed a heatmap of the included viruses and cytokine responses.

### The Cytokine Response to SARS-CoV-2 Sits in Between the Ones Given to Other β-coronaviruses and Influenza A Viruses

**Figure 3:**
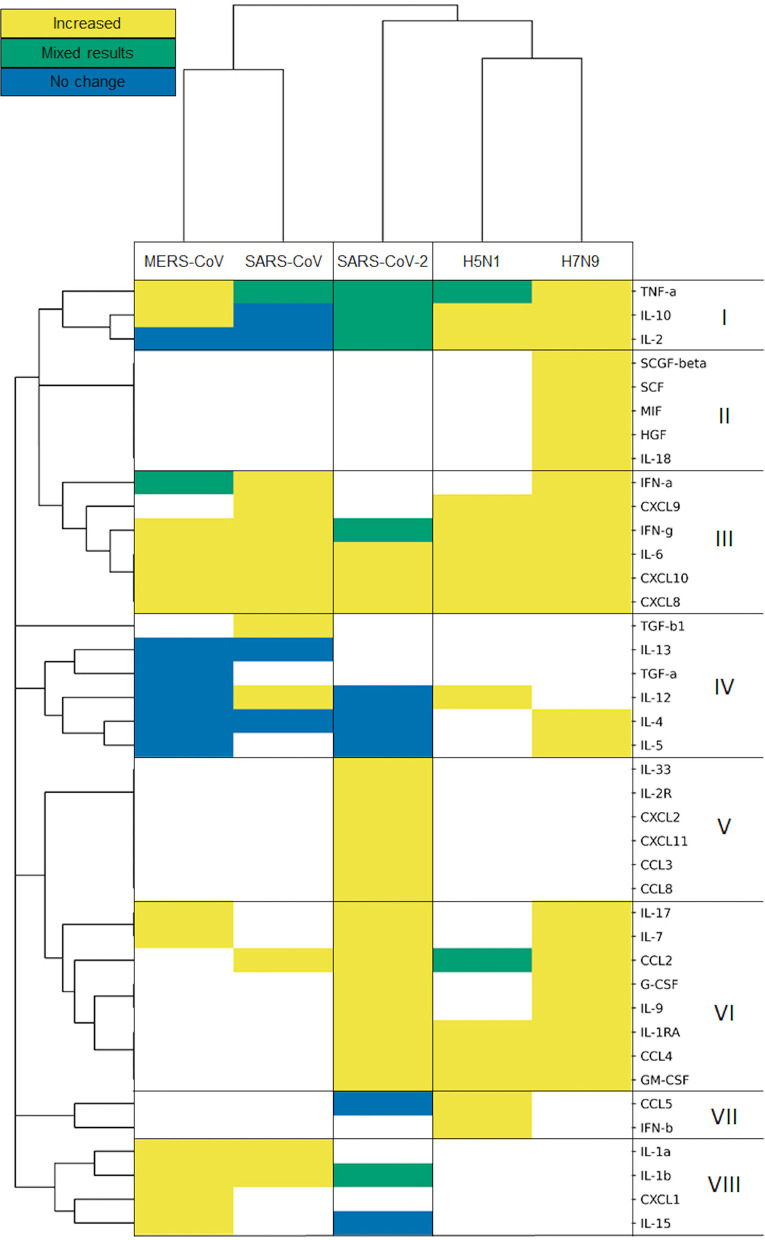
Influenza viruses, SARS-CoV and MERS-CoV and SARS-CoV-2 form separated clusters (I–VIII) based on their cytokine response. Hierarchical clustering is based on Jaccard distance and complete linkage.

We used a hierarchic clustering algorithm on the viruses using Jaccard distance and complete linkage, clustering them based on the cytokine responses they cause. The method groups the pathogens in three clusters. SARS-CoV and MERS-CoV comprise the coronavirus cluster, and H5N1 and H7N9 form the influenza cluster, while SARS-CoV-2 sits in an individual cluster (Figure 3), slightly closer to the two influenza A viruses than to the two β-coronaviruses.

The cluster analysis of cytokines defines eight clusters, based on the direction of their modulation upon infection with each virus. It is important to note that the results of this cluster analysis are biased by the missing information for some cytokines. Bearing this in mind, it is worth looking into the detailed patterns of cytokine responses of the various CRS-inducing viruses. The cytokine cluster I includes the pro-inflammatory, TNF-α, and two anti-inflammatory cytokines, IL-2 and IL-10. All of them had mixed results in SARS-CoV-2, while encompassing all three categories of results for the other two coronavirus infections, which were predominantly increased during influenza infections. Unfortunately, cluster II seems to be restricted to cytokines measured only in H7N9-mediated infections, preventing us from comparing influenza A viruses vs. with β-coronaviruses. Clusters III and VI carry the generally increased pro-inflammatory cytokines, which are elevated for almost all of the viruses but not measured in all of the cases of cluster VI. Among those cytokines are IFN-α and IFN-γ, typical representatives of the general antiviral response (type-I and type-II IFNs), as well as IL-6, one of the most prominent pro-inflammatory cytokines, along various chemokines. Cytokines from Cluster IV measured during coronavirus infections do not fluctuate, while most of them are elevated during an influenza infection, e.g., IL-4 and IL-5 upon H7N9 infections. IL-4 is involved in Th2 differentiation, and the Th2 cells can produce IL-5 to mitigate eosinophil infiltration (53). Such differences observed between virus-specific pathologies reflect the strong alterations observed in coronavirus infections, particularly SARS-CoV-2 (54). The cytokines in Cluster VII and VIII do not always respond to SARS-CoV-2: IL-15 and CCL5 (RANTES) are not elevated after SARS-CoV-2 infection. IL-15 is involved in natural killer cell differentiation as part of an antiviral response (55). Meanwhile, CCL5 mediates eosinophil infiltration which is considered to be involved in the recovery after SARS-CoV infection (56). Clusters II and V contain cytokines measured only in H7N9 and SARS-CoV-2, respectively, whereas TGF-β1 was measured only in SARS-CoV studies in cluster IV.

### Type-I IFN Signaling Can Be More Strongly Altered Upon Infection With SARS-CoV-2 Than in SARS-CoV- or MERS-CoV-infections

Both type-I and type-II IFNs play an instrumental role in the immune response to viral infection.

Our analysis indicates that early induction of type-I IFNs occurs upon H5N1 and H7N9 influenza A infection as well as upon the β-coronavirus SARS-CoV and MERS-CoV (21, 34, 57). However, type-I IFN response is only weakly elicited following a SARS-CoV-2 infection, if at all (37, 58).

**Figure 4:**
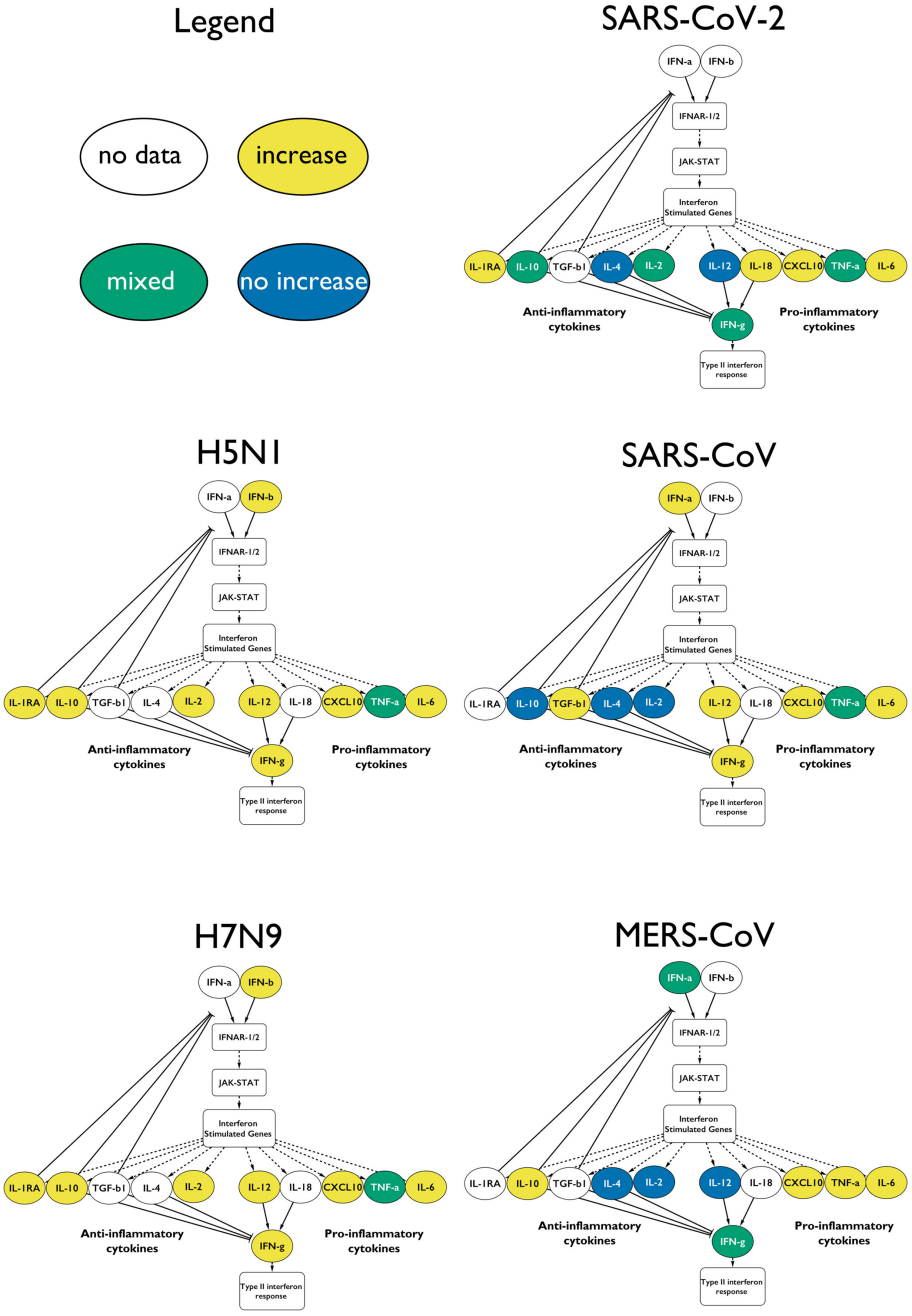
Type-I IFN response upon infection with the different CRS-causing viruses. The measured cytokines in the influenza virus infected patients are increased. In the case of the coronaviruses, the responses are mixed, and not all of the anti-inflammatory cytokines are elevated. Only a fraction of the cytokines is depicted for clarity: yellow for increase in that virus, green for mixed results and blue for no change.

Infection with either of the two influenza subtypes seems to increase the levels of measured type-I IFN-relevant cytokines, resulting in an antiviral immune response, with the appropriate cytokines showing elevated levels in all influenza A studies (Figure 4, Supplementary Table 1).

The β-coronavirus-mediated responses show a much more variable IFN response: with SARS-CoV, we see that the type-I IFN response is active, including the downstream-activated IL-12 that reflects the involvement of mature dendritic cells. IL-12 also indirectly activates IFN-γ further downstream. IL-10 is not elevated, which potentially prevents the downregulation of the type-I IFN response.

In MERS-CoV infections, the type-I IFN response is induced, but not in all cases (59). In some studies, the levels of IL-12 do not increase, in agreement with IFN-γ also staying at low levels. Yet, we see the involvement of the (mostly) anti-inflammatory IL-10. However, caution needs to be applied when looking at IL-10 in an inflammation context, as more and more clinical evidence suggests that this cytokine displays pro-inflammatory characteristics *in vivo* (60, 61).

We showed here that SARS-CoV-2-mediated infections are characterized by a clear dysregulation of type-I IFN response and, consequently, the downstream cytokine signatures, such as IL-4, IL-12, IL-2, and IL-10s, and the downstream type-II IFN response (Figure 4).

## Discussion

In this study, we analyzed relevant cytokine levels measured in patients, each infected with one of the five major respiratory viral pathogens, through a comprehensive literature curation of the published patient data. We generated a map of such responses to help specialists identify routes of interventions to successfully alleviate CRS in different diseases and evaluate whether they could be used in COVID-19 cases. Based on our literature curation, the five investigated viruses cause atypical cytokine responses in severely ill patients, reported here in Figure 3.

While most studies have focused on clinical or phylogenetic parameters (virus genome, patient age, transmissibility, fatality rate, creatinine, and coagulation among others), we aimed to add a mechanistic understanding to the host immune response. The cytokine response during viral infection is a dynamic process, with multiple changes in the cytokine levels during the course of the infection (62). During SARS and MERS infection, a slow initial innate immune response accompanied by the infection of alveolar macrophages leads to increased severity of these lower respiratory tract diseases (63–66). In contrast, SARS-CoV-2 seems to induce a number of cytokines at a very early stage, possibly explaining why the symptoms of severely ill patients deteriorate rapidly (67). A long-lasting pro-inflammatory cytokine production results in high mortality due to the development of severe conditions such as acute respiratory distress syndrome (ARDS) or acute lung injury [9.5% fatality rate for SARS and 34.4% for MERS compared to 2.3% for COVID-19 (43)].

Severe SARS patients show particularly low levels of the anti-inflammatory cytokine IL-10 (Figures 3, 4) (68). During MERS infection, patients develop an expected increased production of IL-10, yet the low levels of IFN-γ-inhibiting IL-4 and IL-2 lead to elevated IFN-γ and the induction of type-II IFN response (Figure 3) (59, 69, 70). In contrast, during influenza A infection, the antiviral response activates without much delay with the presence of an intact negative feedback loop. Both viruses considered in our curation induce most of the pro- and anti-inflammatory cytokines downstream of type-I IFN response (Figure 3). Although influenza A viruses have effectors that dysregulate IFN-I (e.g., NS1, PB1-F2, polymerase proteins), the IFN-I response is nonetheless sustained, and its excessive activation during severe illness can lead to increased mortality. Furthermore, during H7N9 and H5N1 severe infections, TGF-β fails to be activated, contributing to increased pathogenicity (71–73). SARS-CoV-2 stands out from the other β-coronaviruses and influenza A viruses, with a highly perturbed response downstream of type-I IFN signaling, as reflected in the poor balance of measured pro-and anti-inflammatory cytokines (Figures 3, 4). Of note, IFN-α was found to be increased (similar to the other viruses) only in one small (*n* = 4) patient study, which did not match our inclusion criteria. Type-II IFN-γ was also only increased in patients placed in intensive care units (ICUs), while it was within normal ranges in other studies (14, 74, 75).

Although the cytokine signaling enabling the reduction of the inflammatory environment is active (Figures 3, 4), both influenza viruses H5N1 and H7N9 can cause CRS. In severe cases of infection, CRS could result from insufficient production of important cytokines such as TGF-β (73). Furthermore, the presence of impaired and less abundant effector CD4+ and CD8+ T cells was found to be a characteristic feature accompanying CRS in those diseases. Finally, monocytes that normally would differentiate from a pro-inflammatory state to an anti-inflammatory state with enhanced antigen presentation activity as the infection progresses remain in a chronic pro-inflammatory activation state, preventing the normal resolution of the host response (16, 76, 77). In future studies, patient-derived data including the size and activation status of innate and adaptive immune cell populations would help increase the understanding of CRS mechanisms in influenza-mediated diseases.

In our study, we found resolution of the pro-inflammatory immune response to be a key difference between coronaviruses (MERS-CoV and SARS-CoV) and influenza viruses (H5N1 and H7N9). Both MERS-CoV and SARS-CoV induce CRS, yet they also appear to impair the normal resolution of the antiviral immune response. In contrast, H5N1 and H7N9 induce high levels of pro- and anti-inflammatory cytokine levels in severe cases, leading to an inflammatory cytokine storm, yet leaving the immune system unimpeded to move toward a general resolution of the antiviral response appears in Figures 3, 4) (36). However, SARS-CoV-2 induction of the CRS is eventually followed by a resolution of the pro-inflammatory responses in 80% of the cases.

One limitation of this study is the lack of anatomical and dynamic dimensions of the cytokine response. Firstly, the set of cytokines measured in the peripheral blood of each patient across the entire disease course or following recovery varied across the studies analyzed. Patients were sampled at different stages of the disease, which further add to the noise observed in the data. Finally, systematic patient-based studies matching our strict curation criteria could not be collected, leaving many gaps in our comparisons (Figure 3, white cells).

While confirming many already reported disease traits, our analysis has highlighted several new features that are shared or different between the viral diseases analyzed, contributing to filling the gap in the understanding of SARS-CoV-2 and other CRS-causing viruses. Blockage of the cytokine response in SARS-CoV-2 infection through IL-6 specific antibody has failed during Phase 3 randomized clinical trial (NCT04320615), even with promising results in earlier stages (78–80), suggesting that further mechanistic investigation of the cytokine storms during SARS-CoV-2 infection will be needed.

The ongoing accumulation of patient-derived large data sets will inform the research community and clinicians of the intricacy of host/virus interactions (81). Systematic reviews such as this study should be part of an iterative process, increasing the resolution of the comparisons listed above, by continuously integrating novel data. Recently published data and literature repositories, such as H2V and LitCovid, can further enhance the effectiveness of this iterative process (82, 83). In this study, we provided an example of this through a literature curation of patient-derived data and a comparative map across CRS-causing β-coronaviruses and influenza A viruses, linking shared or specific changing cytokines and interferon signaling alterations to those pathogens. In this study, we provided the methodology and scripts to perform this iterative analysis easier in the future.

## Conclusions

Using our literature curation workflow, we showed that based on available patient data, SARS-CoV-2 generates a different cytokine response compared to other CRS causing respiratory viruses. SARS-CoV-2 does not elevate all of the expected cytokines in patients as the other studied respiratory viruses, e.g., the cytokines following an influenza infection such as IL-2, IL-10, IL-4, or IL-5. Although for a subset of pro-inflammatory cytokines, SARS-CoV-2 does induce a similar response to the compared viruses, the literature reports conflicting results for a few important cytokines such as IFN-γ and IL-1β. Applying the collected data to the type-I IFN cascade, the cytokine signature indicates a dysregulation of this process and that of the downstream type-II IFN responses, involving cytokines such as the aforementioned IL-10, IL-2, IL-4, or IL-12.

In our systematic analysis, we collated a map of patient-derived cytokine responses given to different CRS-causing viruses. Our goal is that such a resource of unique and conserved cytokine responses will aid specialists to identify interventions that can alleviate serious cases of COVID-19 and other illnesses that cause CRS.

## Data Availability Statement

The original contributions presented in the study are included in the article's Supplementary Material and available at https://github.com/korcsmarosgroup/CRS.

## Author Contributions

MO and IH collected and analyzed the literature data with DM, and wrote the manuscript together, with contributions from AT, MP, LG, and CS-L. DM performed the clustering analysis for Figure 3. TK supervised the project. All authors discussed the results and contributed to the final manuscript.

## Conflict of Interest

The authors declare that the research was conducted in the absence of any commercial or financial relationships that could be construed as a potential conflict of interest.
